# *“I was so worried”*: Experiences of parents whose infants were admitted to a neonatal care unit

**DOI:** 10.1371/journal.pgph.0004741

**Published:** 2025-06-06

**Authors:** Nicholas Twijukye, Rose Chalo Nabirye, Julius N. Wandabwa, Mary Aleni, David Mukunya, Joshua Epuitai

**Affiliations:** 1 Department of Nursing, Busitema University, Mbale, Uganda; 2 Department of Obstetrics and Gynaecology, Busitema University, Mbale, Uganda; 3 Department of Nursing and Midwifery, Muni University, Arua, Uganda; 4 Department of Public Health and Community Health, Busitema University, Mbale, Uganda; PLOS: Public Library of Science, UNITED STATES OF AMERICA

## Abstract

Parents of infants admitted to a neonatal unit tend to experience emotional stress following admission of their infants in a neonatal unit. The study aimed to explore the experiences of parents whose infants were admitted to a neonatal care unit. We conducted a qualitative study based on strategies of the phenomenological method of inquiry. Participants were recruited at discharge at the neonatal unit. We obtained ethical clearance from the relevant authorities and analysed the data using Braun and Clarke’s thematic framework. We interviewed ten parents (nine mothers and one father). Two themes were identified from the study: 1) sources of parental stress and 2) moderators of parental stress. Parents experienced emotional stress from fears related to the survival chances of their infants, disrupted domestic, economic duties, and challenges meeting the unexpected expenses during hospital admission. Admission to the neonatal unit was perceived to be stressful for parents due to the unfamiliar experience in the neonatal unit, challenges of space, inadequate social amenities, heat from the machines, and restrictive infection and prevention practices. Positive interaction and communication with the healthcare providers, improvement in the infant’s condition, and the ability to adapt and adjust were perceived to moderate parental stress. Parents of infants admitted to a neonatal unit experienced stress for different reasons. Prioritising the psychosocial needs of parents through the development of family-centered care models and support programs is critical in improving the experiences of parents of infants admitted to neonatal units.

## Background

Admission of small and sick newborns to neonatal units in low and middle-income countries (LMICs) ranges from 5% to 79% [1]. The wide variation reflects the difference in the levels of healthcare facility settings, hospital protocols, and reasons for neonatal admission in these LMICs [1]. Complications during pregnancy, childbirth, and or postnatal period may result in the admission of infants to a neonatal unit [2]. The leading causes of neonatal unit admission are complications of prematurity, perinatal asphyxia, and neonatal sepsis [3–5]. In sub-Saharan Africa, the mortality rates of infants admitted to neonatal units range from 2.8% to 22.5% [1].

Parents of infants admitted in neonatal units tend to experience negative emotional and physical consequences following the admission of their infants in neonatal units [6,7]. About half (40–50%) of parents with infants in neonatal units experienced clinical depression, anxiety, and post-traumatic stress disorder [7]. In Australia, three months after discharge, mothers of infants admitted to neonatal units had 21% generalized anxiety disorder, 8% major depressive disorders, and 6% post-traumatic disorders [8].

The sources of stress and anxiety among parents stem partly from restrictive visitation policies, high direct and indirect hospital costs, and inadequate maternity leave policies [9]. Furthermore, the negative emotions emanated from fear of unfavorable outcomes and feelings of guilt following premature births [2,6,7]. Early parental-newborn separation, quality of interaction with the healthcare workers, neonatal environment, the fragile state of small and sick newborns, and the painful invasive procedures further provide additional sources of stress for the parents [10].

Parents’ experiences following admission in neonatal units play a substantial role in improving neonatal care [11]. This is especially true in LMICs where the burden of high-risk newborns is high amidst resource constraints [1]. Providing a better understanding of parents’ experience in neonatal units can open a window for integrated programs, policies, and efforts to create a more holistic and supportive environment within the neonatal unit setting [12]. Therefore, the aim of conducting this study was to explore the experiences of parents whose infants were admitted to a neonatal unit.

## Materials and methods

### Ethics statement

We obtained ethical approval from the local Research Ethics Committee (Ref: 2023-88), and administrative approval from the hospital. The study setting was kept confidential given the sensitivity of the study findings. All participants were enrolled after obtaining written informed consent, while the Belmont ethical principles of beneficence, non-maleficence, justice, and respect of persons were followed.

### Study design and setting

We conducted a qualitative interview study borrowing upon the phenomenological strategy of inquiry. The design was appropriate for exploring the experiences of parents whose infants were admitted to a neonatal unit. This study was conducted at a public health facility at the level of a Regional Referral Hospital in a low-income country in sub-Saharan Africa. The facility supports several lower-level, government-run health centers and private not-for-profit health units. The neonatal unit is at the level two neonatal care unit and accepts neonatal admissions, totaling over 2,000 annual neonatal admissions, from various sources including district hospitals, health centers, private clinics, and home deliveries. The neonatal unit has a dedicated area for caring for severely ill infants and a room for kangaroo mother care. The neonatal unit in the hospital provides services related to temperature support, phototherapy, oxygen therapy, feeding support (including nasal gastric tube feeding), and parenteral administration of essential drugs. The neonatal unit has healthcare workers of different levels including a neonatologist, nurses, medical doctors, and allied health professionals. However, the unit does not have dedicated mental health professionals or social workers to provide social and psychological support services for parents in the unit.

### Sampling procedure

We purposively sampled parents of infants who were admitted to the neonatal unit. Purposive sampling was used to select participants who could provide a rich in-depth description of the phenomenon under study [13]. We purposively selected infants with diverse diagnoses including those who were admitted following complications of preterm birth, low birth weight, birth asphyxia, and jaundice. The sample size for the study was guided by the principle of information power [14]. Information power refers to a process where the sample size is determined by the quality of data collected in the study [14]. Consequently, a sample with rich information may require fewer study participants [14]. Sufficient information power depends on the study aim, the specificity of the sample, the use of a theory, the quality of interviews, and the analysis strategy [14]. Sufficient information may be realized with smaller sample sizes when the study aims are narrow, the study uses an established theory and the study participants possess the phenomenon of interest [14]. Furthermore, sufficient information power is attained with fewer participants when interviews generate in-depth information and when the analysis strategies focus on providing an understanding of typical instead of diverse perspectives [14]. Our study aim was specific to parents’ experiences in a neonatal unit, while we purposively selected study participants with such experiences. The study was based on the phenomenological underpinnings while the depth of interviews, and the thematic analysis strategy underscore how our study aligned with the tenets of information power [14]. Furthermore, pragmatic and contextual factors tend to determine the study sample sizes in qualitative studies [15]. We had sufficient information power when we had 10 in-depth interviews of which no new insights were identified from the interviews.

### Data collection tool and procedure

Data were collected from 01st October 2023 to 30th November 2023. Data were collected using in-depth interviews with parents of infants admitted to neonatal units. The parents were recruited at discharge and interviewed in a quiet and private room without non-study participants present. A semi-structured interview guide was used for data collection. The interview guide was developed from previous studies regarding parents’ experiences in neonatal care [16]. The interview guide had questions to explore experiences related to initial feelings on admission, experience with caring for infants in the neonatal unit, interactions with the neonatal health care team, decision-making concerning the care and treatment options, the challenges and coping mechanisms during the stay, the extent of involvement in the care for infants, and suggestions changes for improved neonatal unit experience for the parents (S1 Text). The interviews were audio recorded after obtaining consent from participants. The first author conducted the interviews in the native language (S1 Checklist). The interviews took an average of 25 minutes with each participant but ranged from 18 to 37 minutes.

The first author approached parents shortly after discharge from the neonatal unit. The first author is a male nursing student fluent in both English and the native language and was currently attached to the neonatal unit at the time of data collection. All the parents approached willingly accepted to participate in the study.

### Data processing and analysis

Data was translated from native languages to English and transcription was conducted verbatim. The first and the last author analysed the data. Data were analysed using inductive thematic analysis and we used ATLAS.ti 9 software to organize the codes [17]. The first step involved reading the transcripts for several times to understand the data [17]. Subsequent steps involved generating initial codes from the transcripts [17]. The third step involved searching for themes including collating the different codes into potential themes [17]. The fourth and fifth steps involved reviewing, defining, and renaming the themes [17]. Lastly, the themes and codes were described in the final report [17].

## Results

### Demographic characteristics of the parents and infants

The study comprised nine mothers and one father who were involved in the caregiving role of infants admitted to the neonatal unit (Table 1). One parent spent less than seven days in the unit, while four spent between 8–21 days, and five spent above 21 days of admission. The infants whose parents were sampled had the following conditions: complications of prematurity, low birth weight and birth asphyxia on admission, jaundice, respiratory distress syndrome, pneumonia, hypoxic_-_ischemic encephalopathy and a congenital heart defect.

**Table 1:** Description of the study participants.

Variable	Frequency (percentage)
**Parents**	
Fathers	1(10%)
Mothers	9 (90%
**Level of education**	
Primary or lower	4 (40%)
Secondary and above	6 (60%)
**Duration of stay in the neonatal unit**	
Less than 7 days	1 (10%)
8-21 days	4 (40%)
More than 21 days	5 (50%)

### Experiences of parents with neonatal admission of infants admitted in neonatal unit

Two themes were identified to represent parents’ experiences. The themes included sources of parental stress and the moderators of parental stress (Table 2).

**Table 2:** Experiences of parents with admission of infants in a neonatal unit.

Codes	Subthemes	Themes
Worries about the infant’s survival chances Early parental separation The poor state of the infant Worsening infant’s condition	Stress from survival fears	Sources of parental stress
Restrictive infection and prevention practices Unfamiliar environment Overcrowding Inadequate social amenities	Stress from admission to the neonatal unit	
Disrupted domestic caring responsibilities Disrupted economic duties	Stress from disrupted domestic duties	
Out-of-pocket expenditure on medicines, supplies, and diagnostic tests Challenges meeting expenses on basic needs	Stress from unexpected expenses	
Self-belief Signs of improvement Acceptance and tolerance of the neonatal environment	Improvement in the infant’s condition	Moderators of parental stress
Respectful interaction Effective communication Timely and responsive treatment and care	Positive interaction with healthcare workers	
Caregiving roles Learning to care for small and sick infants Decision-making roles Prioritizing the needs of infants	Adjusting and adapting to the neonatal unit	

### Theme 1: Sources of parental stress

#### Subtheme 1: Stress from survival fears.

Several parents experienced worries and fears related to the survival chances of their infants. Parents feared and worried about whether their infants would survive. The poor state of the infant’s condition especially their small stature added to the stress. The uncertainty of the infant’s outcomes created anxiety and helplessness throughout the time of admission.

*“It made me sad because my baby was premature and I was so worried because I thought my baby was going to die. I was worried because I gave birth at home…. I came here not expecting the baby to be alive because he got a lot of coldness ”* (Mother, 31 years; caring for a preterm infant with low birth weight).

In some cases, the feelings of anxiety set in at the possibility of a preterm delivery, through the laboring process till admission of the infants to the neonatal unit.

*“So, it was not even [time], and I don’t even expect her to deliver at that time.…they told me that……we are now waiting for the time to deliver. So, I was stressed because…I had not even prepared to receive a baby. So, I got stressed that day.”* (Father, 35 years; caring for a preterm infant with jaundice).

Postpartum mothers who had a Caesarean section delivery and were admitted to a separate postnatal ward could not fully be with their infants in the neonatal ward. These mothers had limited interaction with their infants and experienced feelings of isolation. The early parental separation from their infants resulted in heightened feelings of uncertainty and worries related to the survival chances of their infants.

*‘I went back to postnatal ward silently and weak but then I felt like I wanted to see my baby. I came back to look at my baby who was very small, [had] breathing difficultly and I thought this one wouldn’t come out into an adult. I was scared that when they gave me food, I had no appetite because I had a worry that any time, they are going to call me that the baby has passed on”.* (Mother, 33 years caring for an infant with very low birth weight).

One mother, who had a Caesarean section, spent five days before joining to be with her infant:

*“The situation hasn’t been good but you leave it all to God. I know it gets easier. The situation we went through, from day one. As I told you, I spent 5 days before I joined him.”* (Mother, 32 years; caring for a preterm infant with low birth weight).

Parents had more worries following setbacks in the healing process and deterioration in the infant’s condition. Some parents contemplated and imagined the worst-case scenarios during this period.

*“They started having hope of sending me home. Unfortunately, the child got infection. Almost he was going [to die]... Now the child stopped breathing. … the morale! I lost it completely…I had lost the morale, imagining of so many things…”* (Mother, 33 years; caring for an infant with very low birth weight).

#### Subtheme 2: Stress from admission in the neonatal unit environment.

Admission to the neonatal unit was a source of stress for most parents. The emotional stress originated from the distressing unfamiliar neonatal standards and the infection and prevention practices. The infection prevention requirements like hand washing, wearing of masks, and prohibition of using casual clothes within the unit were identified to cause additional stress for parents in the neonatal unit. Restrictions in the number of visitors and the persons allowed in the neonatal unit made it more difficult for parents to share caregiving roles. This meant that a limited number of caregivers would be allowed in the unit at a time. Restricting the number of caregivers in the unit was especially challenging for mothers who were already frail and were trying to recover from the childbirth experience.

*“…they don’t want visitors inside here because they are sure those visitors can bring diseases to babies. Now, when you have such a baby, you are supposed to be strictly two people. …[but] they don’t need more than one caregiver. So, things were hard for me in that way.”* (Mother, 33 years; caring for an infant with very low birth weight).

Parents who struggled to adhere to these infection and prevention practices such as wearing masks experienced negative interaction from the hospital staff.

*“Some of our healthcare staff, they are very harsh to us. They can chase you! Why don’t you have a mask? You go away….There is a lot of stress when you are inside here…”.* (Mother, 24 years, caring for a preterm infant with low birth weight).

The neonatal environment itself was new, unfamiliar, and unsettling for the parents. The sight of infants on oxygen, and the sight of other infants passing was distressing for the parents.

*“Too much worry. Because the environment was new to me. I’d never seen such before. I’d never seen children on oxygen. I see babies die all the time, all the time, all the time. And sometimes I get worried that, ah, will this one of mine survive?”* (Mother, 33 years; caring for an infant with very low birth weight).

Parents recognised the need to monitor and be with their infants throughout neonatal admission. This made it difficult for the parents to leave their infants alone to go for food, while the restrictions to bring food into the unit made some parents go for long hours without food. Parents narrated about the overcrowding in the unit, the heat from the machines, and the sleeping difficulties amidst balancing their emotions with caregiving responsibilities. Parents narrated standing throughout the day and night. This was related to the limited resources such as insufficient beds for infants, restrooms, and facilities for sitting and sleeping. Altogether these experiences made it difficult for parents to care for their infants in the neonatal unit, while some parents had physical and mental health problems including swelling of legs and mental exhaustion.

*…. It wasn’t easy because inside here… it’s really hot. Secondly, you don’t get a place to sleep, and thirdly food they don’t allow anything to be brought in. Now, all of that is not easy.”* (Mother, 32 years; caring for a preterm infant with low birth weight).

One parent narrated the overcrowding in the unit and how it could cause cross-infection:

*“I see the beds we are crowded together, one small bed and we put about four babies. This child’s disease can infect another eeeh the beds are not enough.”* (Mother, 31 years; caring for preterm infant with low birth weight).

#### Subtheme 3: Stress from disrupted domestic duties.

Concerns for other children left at home, the family, and other responsibilities at home were also a source of emotional distress to most of the parents. It contributed to the persistent anxiety and emotional strain throughout the hospital stay.

*“The other children, I think they miss seeing dad and mom. I’m sure they miss us because they stay with the grandmother. Now I don’t even know what they are, what they ate because I’m so far. It makes me feel bad; I want to see them. I live in worry, that what if my children are sick, whether they have eaten what there at home”.* (Mother, 26 years; caring for a preterm infant with low birth weight).

Parents described that neonatal admission disrupted them from executing their job activities and domestic duties. This resulted from prioritising the child’s health amidst the challenges of balancing work and parenting responsibilities. Domestic duties were mostly disrupted during this time of admission.

*“After all, jobs, etc. are ruined but I don’t mind because my son is fine and alive.”* (Mother, 31 years; caring for a premature infant with low birth weight).

Neonatal admission disrupted social roles and responsibilities. Mothers were tasked with the caregiving roles, while fathers provided financial and emotional support.

*“I have been here for a long time. I left home when I had grown my food there and now, I don’t know what it is like right now. If you are not there you cannot take care of them, you put the work aside and don’t care much about it. The money, the baby’s father goes and works and supports us, mother and child.”* (Mother, 19 years; caring for an infant with birth asphyxia).

#### Subtheme 4: Stress from unexpected expenses.

Parents acknowledged receiving free medical services including consultation, support, and treatment for their infants in the neonatal unit. Medicines and other therapies were availed and administered at a free cost to everyone during the hospital stay. However, some parents described instances of unexpected expenses on specific drugs and supplements which were not available within the unit and they had to be bought from private pharmacies.

*“They may tell you that there is no medicine and they have to prescribe for you to buy it and you don’t have money.”* (Mother, 34 years; caring for twin infants with very low birth weight).

A parent narrated how costs related to acquiring food outweighed all the other expenses during admission. Besides, medicines and food, there were other expenses on small items like diapers, and infant clothing among others which contributed to the financial strain throughout the hospital stay.

*“I’ve lost a lot. I’ve used a lot of money just buying some other small, small things. Like when you feel like to take some water, you go and buy water. So, in the village there, we don’t buy water. So, I realized from here that you have to buy even water.”* (Father, 35 years; caring for a preterm infant with jaundice).

Some parents experienced financial hardships in covering for the many financial demands during the hospital stay. Some parents acquired financial assistance from the infants’ fathers, friends, and relatives while the little savings could not sustain them during the admission. Most parents resorted to selling mainly agricultural products to raise money to cater for neonatal admission.

*“Even acquiring money is not easy. We farm and get something to eat or I sell my beans or vegetables and get some money to support myself.”* (Mother, 31 years; caring for a premature infant with low birth weight).

### Theme 2: Moderators of parental stress in the neonatal unit

#### Subtheme 1: Improvement in the infant’s condition.

Most parents experienced initial worries and doubts upon admission. However, emotions evolved from fear to hope as the infants’ conditions improved:

*“At first, I was worried because I did not have hope that the baby would be treated and become fine. But with time as the child was improving, I became comforted”.* (Mother, 32 years; caring for a preterm infant with low birth weight).

Parents expressed feelings of relief and gratitude during the whole recovery process of the infant, especially with signs of overall improvement such as weight gain, effective breastfeeding, and the reduced need for oxygen. These positive emotions were tied to the positive changes and improved health and well-being of the infant from a critically ill state up to discharge from the neonatal unit. Parents were also grateful for the support they received in the neonatal unit.

*“I felt good because he has found peace, he has received good treatment and he has found life, he has no problems now. It made me feel good because they saved my child’s life. I have never had any problems in the time I have been there. They treated my son well, everything is okay”.* (Mother, 23 years; caring for a preterm infant with low birth weight).

Parents felt good when their infant’s condition improved because most of them did not expect their infant’s condition to improve:

*“But when I got here the health workers welcomed me and treated my baby and now, I feel so happy because they have sent us home. We are both going home alive which I did not expect.”* (Mother, 31 years; caring for a premature infant with low birth weight).

The belief in God acted as a source of solace during the hospital stay. The recognition of the day-to-day positive changes in the infants’ condition kept the parents hopeful during admission.

*“You have to endure as a person because I know what brought me here. As long as the baby has come out safely. I thank God because being here is not forever, you come and they treat the baby, they discharge you away and you go back.”* (Mother, 32 years; caring for a preterm infant with a heart defect).

Some parents experienced positive feelings on admission to the neonatal unit because they perceived it as crucial for the survival of their infants. The positive emotions emanated from confidence in the services provided, and assurance that their infant would survive.

*“I felt okay. Yeah, because I knew my baby is going to survive, I knew he is going to get all the services from here”.* (Mother, 24 years; caring for a preterm infant with low birth weight).

Parents acknowledged that admission to the neonatal unit was a good thing for their infants. Because the neonatal unit was different from a home setting, it helped some of the parents to minimize the challenges they experienced while in the neonatal unit.

*“As a person, it hurts, but you are brave because you want the child’s life. That’s what makes you more patient…It makes me feel better when you’re here; it’s better than when you’re at home.”* (Mother, 31 years; caring for a preterm infant with low birth weight).

#### *Subtheme 2*: *Positive interactions with healthcare providers.*

Parents reported positive interactions with the staff during the hospital stay. The positive experiences with the healthcare professionals were perceived in terms of proper care and treatment for their infant in the neonatal unit.

*“Our doctors and nurses are nice. They take care of us even if the room is hot. They live with us. They never leave us. They take good care of our children. Even if the child’s condition changes, they are almost available treating us well, so I haven’t seen a doctor treat us badly.”* (Mother, 31 years; caring for a preterm infant with low birth weight).

Healthcare providers were a source of encouragement and support throughout the admission time with positive sentiments towards the nursing staff.

*“Whenever the condition changes, the nurses, the doctors, they check the baby all the time. They give treatment without any payment. They have been very good people. They encourage you, and they tell you the child will be fine. They love my baby all the time.”* (Mother, 32 years; caring for a premature infant with low birth weight).

Another parent described the interactions as good in that the healthcare professionals did not threaten, were unbiased, and did not segregate against them. The healthcare providers were noted to treat everyone equitably with collaborative efforts in the care of infants. The health workers were determined to provide good care, support, and guidance toward the well-being of infants as a common goal:

*“Because they are not even threatening us, they just do their part. They don’t segregate. That was my happiness. Now, there is that tube, eh, used for feeding. So, the baby used to, to remove it. So, when you go there, the nurse might tell you that, ah, no, let us put another one. Don’t mind! Don’t mind! So, they used to help me.”* (Father, 35 years; caring for a premature infant with jaundice).

In contrary, a mother narrated a form of interaction described as bad where the healthcare workers treated them harshly in the form of insults without assisting them on time. Mistreatment was experienced when parents asked for help in case the infant had a problem.

*“Of course, they are very harsh to the mothers. Sometimes you find the baby has pulled out the nasogastric tube or the canula. But when you try to tell them, at times they can be harsh…”* (Mother, 24 years; caring for a preterm infant with low birth weight).

One parent noted that the mistreatment occurred often at later times of the day when the staff got exhausted.

*“And during evening time, the time they come, they are already tired. And when they are tired, they are tough. When they are tough, mostly they give attention to these babies who are just coming in.”* (Mother, 33 years; caring for an infant with very low birth weight).

#### Subtheme 3: Adjusting and adapting to the neonatal unit.

Parents adjusted and adapted to the neonatal unit by participating in the caregiving roles. The caregiver roles included active involvement in basic care responsibilities of the infant which ranged from feeding (breastfeeding and or feeding using expressed milk), maintaining good hygiene, and routine tasks of changing diapers and clothing of infants. One parent mentioned that caregiving roles included kangaroo mother care and ensuring that the infant received the prescribed medication in the right time.

*“Also, I do wash his clothes and so on. Keep watch and care after him during the day and night, give him milk in time after 2 hours, and chest contact. I have to make sure I take the file and the baby receives medicine.” (*Mother, 32 years; caring for a preterm infant with a heart defect).

The parents admitted to having gained new and more knowledge related to caring for their infants as the healthcare professionals advised and guided them. This was mainly for the small infants that had special caring requirements.

*“Now I have learned how they care for a small baby, how do you hold it, so the way you treat it is not like these nine-month-olds.”* (Mother, 19 years; caring for an infant with birth asphyxia).

Parents described that decision-making concerning the infant’s care was limited to monitoring the infant’s condition and calling for help immediately from the healthcare staff in case of any problem. The parents entrusted healthcare personnel with the authority to make decisions and plans concerning treatment given their lack of expertise in healthcare matters.

*“The part I take is me just to monitor the baby, How the baby is doing, in case the baby is not in a good situation, I call them and they find out what should be the problem.”* (Mother, 32 years; caring for a preterm infant with low birth weight).

Prioritizing the health needs of infants ahead of their own helped parents to withstand the challenges in the neonatal unit. Challenges of lack of sleep, rest, and swollen legs were not of concern to most parents as long as the infant’s condition improved.

*“Because of standing and sitting, you don’t even rest. You don’t sleep….. The legs started swelling me also. So, at the same time, I was…a patient…I just continue with the situation because I want to see that my baby will be okay. So, I was minding of my baby only. I don’t mind on my side”* (Father, 35 years; caring for a premature infant with jaundice).

Another parent exemplified how they prioritized the health needs of their infant despite the challenges in the neonatal unit:

*“Life is not easy but because you want the life of a child. Heat in the room, sitting in one position from morning till morning,…. I don’t see anything wrong because my child’s life is far better.”* (Mother, 31 years; caring for a preterm infant with low birth weight).

Parents described that they had to adjust to challenges as part of the experience with patience and determination due to their desire for the infants’ lives. Adaptation to infant care involved techniques such as milk expression and chest-to-chest holding of the infants. Additionally, acceptance of admission in the neonatal unit as being a better environment for an infant compared to home gave them reasons to be patient and more resilient.

*“…So, I came to learn that when you are in this place, you make sure by 6 am you run out very fast. You bathe, you take tea. By 7:30 am, you enter before our boss comes. So, when I got that experience a bit, I changed at least life.”* (Mother, 33 years; caring for an infant with very low birth weight).

Adjusting to the neonatal environment also included knowing the schedules, policies, and practices in the unit. Parents devised mechanisms to abide by the infection and prevention practices in the neonatal unit through measures such as waking up early to clean up and prepare before the ward rounds start.

*“In fact, the issue of locking us outside, you wake up at around 5:00am then you go out…So you time that early morning when the door is still open, you go out, you take a shower and come back. Then you remain now inside.”* (Mother, 24 years; caring for a preterm infant with low birth weight).

## Discussion

The study explored the experiences of parents following the admission of their infants to a neonatal unit. Our study noted negative emotional responses to admission characterised by feelings of fear, anxiety, and helplessness. Admission to neonatal resulted in worries over the survival chances of their infants, unexpected expenses financial strain, and disruption in domestic duties amidst challenges in the neonatal environment. These results are important to significantly enhance the quality of care provided in the neonatal and support parents in their vital role as primary nurturers for their newborns in low and middle-income settings.

Our study highlights experiences of stress, anxiety, sadness, and helplessness among parents following admission to the neonatal unit. A scoping review highlighted a high (40–50%) prevalence of stress, anxiety, depression, and posttraumatic stress noted among parents of infants admitted to neonatal units [7]. Consistent with other study findings [7,18,19], parents tend to experience a high burden of fear, anxiety, constant worries, loneliness, and self-doubt following the admission of their infants to neonatal units. In some cases, the emotional feelings tend to start from the impending pre-term birth, through the laboring process to the point of neonatal admission. A scoping review showed that about 30–60% of parents continue to experience emotional symptoms even after discharge from the neonatal unit [7]. Like in other studies [6,12,18], the constant worries were related to fears of the survival chances of their infants, seeing other people’s infants die, and the worsening condition of the infant. Parents were optimistic following some indication of improvement in the condition of the infant, which further underscores the critical role of the survival of infants on the mental health of parents in a neonatal unit.

In our study, restrictive infection and prevention policies were implemented in the unit to prevent hospital-acquired infections, a finding that was reported in other settings [18,19]. Restricting the number of parents in the unit, and barring of visitors and food in the unit was seen to lead to isolation, loneliness, and exhaustion among parents. Exhaustion among parents occurred from lack of food, sleep, and standing for longer hours caring for the infants. Although healthcare providers limited the number of parents in the unit to prevent nosocomial infections, the continuous presence of parents in the neonatal unit reduced neonatal sepsis by 18% but also reduced the overall neonatal mortality by 25% in other settings [20,21]. This underscores the importance of the mother-newborn care unit which integrates the continuous presence of caregivers in the neonatal unit [20,21].

The neonatal environment was new, unfamiliar, and unsettling for the parents, a finding that was consistent with other studies [22,23]. In our setting, the neonatal was small, hot, crowded, and it had a shortage of beds which resulted in the sharing of neonatal beds. Parents were concerned about cross infections from crowding, while in some settings crowding was linked to a fears regarding theft and swapping of infants in crowded units [17]. These parental fears may cause reluctance for their infants to be admitted in neonatal units [19]. Parents experienced mental exhaustion, lack of sleep, and swelling of legs from lack of seats and beds in the unit, inadequate social amenities like restrooms, the unsettling machines and witnessing neonatal mortalities.

Our study was conducted in a public facility that offers free medical care services to all patients [17]. However, parents, experienced financial strain related to unexpected expenses on direct costs from out-of-pocket expenditures for medicines, supplies, and diagnostic investigations which were unavailable, and indirect costs on food and other welfare concerns. Although parents cared more about the survival of their infants [17], the financial constraints led to reluctance for continued admission to the neonatal unit [22]. Shortage of essential medicines, supplies, and services (e.g., diagnostic investigations) requiring parents to buy which some of them could not afford, may have affected the quality of care offered to them.

Neonatal care requires almost full-time attendance to be able to sustain provision of caregiving roles [24]. Consistent with previous studies [19,22], our study noted disrupted domestic and economic responsibilities during admission to the neonatal unit. Women were unable to attend to the children left at home or engage in income-generating activities. The disrupted domestic, social, and employment duties added to more stress for the parents, but it may have also reduced their ability to raise more funds to pay for hospital bills.

Parents of infants admitted in the neonatal unit require adequate support from healthcare providers [25]. Healthcare providers’ adequate support, rapport, and effective communication can empower parents to actively participate in the care of their infants, especially in performing procedures such as skin-to-skin contact where parents may be reluctant, while it can also reduce emotional stress and ease the transition to parenthood [25]. Our study noted variable interpersonal relations among healthcare providers which ranged from respectful, supportive care to disrespectful and harsh treatment of parents. Similar studies have cited communication gaps where healthcare providers seemed to ignore parents [22,26]. The staffing shortages, heavy workload, poor remuneration, and the need to comply with regulations in neonatal may account for the existing communication gaps and poor interpersonal relations with the parents [19]. The healthcare providers may also be displacing their frustration to the parents as a result of work burnout and poor mental health suffered from work in the neonatal unit [7,18]. Addressing the existing interpersonal relations, and communication gaps between parents and healthcare providers may improve parents’ experiences, foster effective relations, and promote positive neonatal health outcomes.

Family-centered care involves active involvement, participation, and collaboration between family members and healthcare workers in caring for infants [27]. Family-centered care promotes parents’ ability to care for the small and sick infants, reduces parental stress, and promotes activities such as breastfeeding and kangaroo mother care that improve overall neonatal well-being [27]. Parents, in our study, were actively involved in the care of the infants. The care involved feeding, monitoring the condition of infants, and ensuring they receive treatment. A similar finding has been reported in Uganda where parents were noted to perform duties such as reminding nurses of treatment schedules [23]. In our setting, parents were reluctant to perform roles designated for nurses, a finding that was similar to what was seen in Rwanda [19]. Implementing a family-centered care model could, therefore, equip parents with competencies to be involved in providing care to their infants in close partnership and collaboration with the healthcare workers [27].

### Study strengths and limitations

Our study provides an insightful description of the experiences of parents following the admission of their infants in the neonatal unit. Interviews were conducted at discharge to enable a comprehensive description of the entire neonatal experience. However, this could have led to recall bias of the initial encounters in the neonatal unit. We did not include parents who lost their infants because of ethical reasons given that interviews were conducted immediately after discharge. Exploring the experiences of parents who lost their infants during admission in the neonatal unit could have provided additional strength to the study. Although we attempted to have fathers in the study, a few were available in the unit as primary caregivers amidst gender norms. The gender imbalance may have limited exploration of diverse perspectives. In addition, the findings may be limited by the lack of the healthcare provider’s perspectives to account for their experiences interacting with parents in the neonatal unit. Further qualitative studies need to be conducted to explore the experiences of parents who lost their infants and the perspectives of health workers regarding the impact of neonatal admission on parents. Future quantitative studies should be conducted to determine the short and long-term impact of admission in neonatal units on parents.

## Conclusion

Parents reported experiencing emotional stress following neonatal admissions. Parental stress was related to fears of survival chances of infants, challenges meeting hospital costs, and disrupted domestic and economic responsibilities. The neonatal environment itself was seen to cause stress among parents especially related to the new neonatal experiences and the restrictive infection prevention practices. Parents made adjustments and adaptations following admission to the neonatal unit. The study provides important insights and policy implications to improve the quality of care for small and sick newborns. Empowering parents in neonatal units nearing discharge to provide peer-to-peer counselling to parents of newly admitted infants may help assuage negative emotional responses. Bridging communication gaps and relationships between parents and healthcare providers, empowering and involving parents in the care of infants, improving social amenities in the neonatal unit, and developing family-centered care policies may improve the experience of parents during admission to neonatal units.

## Supporting information

S1 Text Interview guide. (DOCX)

S1 Checklist Consolidated criteria for reporting qualitative studies (COREQ): 32-Item checklist. (DOC)
