# Supplementary material for: “I was so worried”: Experiences of parents whose infants were admitted to a neonatal care unit
Source: PLOS Glob Public Health. 2025 Jun 6;5(6):e0004741. doi: 10.1371/journal.pgph.0004741 (PMC12143576; doi:10.1371/journal.pgph.0004741)
Supplement: S1 Text — (DOCX) [file pgph.0004741.s001.docx]

**INTERVIEW GUIDE**

1. How did you feel when your baby was admitted to the neonatal unit (NNU)?
2. Please, tell me about your experience with caring for your baby with in the NNU? /
3. How has the NNU admission experience been for you as caretaker?
4. Tell me about your interactions with the NNU healthcare team?
5. How were decisions about your baby’s care and treatment options made?
6. What were your main challenges and coping mechanisms during the baby’s stay in

the NNU?

1. To what extent were you involved in your baby’s care while in the NNU?
2. How did you maintain a connection and bond with your baby during the NNU
3. stay?
4. Finally, are there any changes you would suggest to improve the NNU experience for caretakers?
