# Supplementary material for: “I was so worried”: Experiences of parents whose infants were admitted to a neonatal care unit
Source: PLOS Glob Public Health. 2025 Jun 6;5(6):e0004741. doi: 10.1371/journal.pgph.0004741 (PMC12143576; doi:10.1371/journal.pgph.0004741)
Supplement: S1 Checklist — (DOC) [file pgph.0004741.s002.doc]

**Consolidated criteria for reporting qualitative studies (COREQ): 32-item checklist**

| **No. Item** | **Guide questions/description** | **Reported on Page #** |
| --- | --- | --- |
| **Domain 1: Research team and reﬂexivity** |  |  |
| *Personal Characteristics* |  |  |
| 1. Inter viewer/facilitator | Which author/s conducted the interview or focus group?  **Response:** The interviewers were identified and described. The following statement has been included: *The first author conducted the interviews were with extensive training and experience in qualitative data collection in the native language.* | Page 5 |
| 2. Credentials | What were the researcher’s credentials? E.g. PhD, MD  **Response:** We have mentioned the credentials of the researcher who conducted the interviews.  *The first author is a nursing student……* | Page 5 |
| 3. Occupation | What was their occupation at the time of the study?  **Response:** We have described the occupation of the interviewer.  *The first author is male nursing student…..who was currently attached at neonatal unit at the time of data collection.* | Page 5 |
| 4. Gender | Was the researcher male or female?  **Response:** Gender has been mentioned | Page 5 |
| 5. Experience and training | What experience or training did the researcher have?  **Response:** We have mentioned the experience of the researchers who did interviews. | Page 5 |
| *Relationship with participants* |  |  |
| 6. Relationship established | Was a relationship established prior to study commencement?  **Response:** The researcher may have interacted with the study participants during clinical attachment in the ward. | Page 5  . |
| 7. Participant knowledge of the interviewer | What did the participants know about the researcher? e.g. personal goals, reasons for doing the research  **Response:** The participants were informed about the personal reasons for doing the study during the consenting process. | Page 5 |
| 8. Interviewer characteristics | What characteristics were reported about the inter viewer/facilitator? e.g. Bias, assumptions, reasons and interests in the research topic  **Response:** The interview’s occupation was described. | Page 5 |

| **Domain 2: study design** |  |  |
| --- | --- | --- |
| *Theoretical framework* |  |  |
| 9. Methodological orientation and Theory | What methodological orientation was stated to underpin the study? e.g. grounded theory, discourse analysis, ethnography, phenomenology, content analysis  **Response**: The study borrowed strategies from the phenomenological inquiry.  *We conducted a qualitative interview study borrowing upon the phenomenological strategy of inquiry……* | Page 4 |
| *Participant selection* |  |  |
| 10. Sampling | How were participants selected? e.g. purposive, convenience, consecutive, snowball  **Response**: Purposive sampling has been described in the section of study participants and sampling methods. | Page 4 |
| 11. Method of approach | How were participants approached? e.g. face-to-face, telephone, mail, email  **Response:** The method of approaching participants have been described. The following statement has been included;  *The first author approached parents shortly after discharge from the neonatal unit.* | Page 5 |
| 12. Sample size | How many participants were in the study?  **Responses:** We mentioned the sample size of the participants in the section of sample size | Page 4 |
| 13. Non-participation | How many people refused to participate or dropped out? Reasons?  **Response:** All the participants accepted to be part of the study.  *All the parents approached willingly accepted to participate in the study.* | Page 5 |
| *Setting* |  |  |
| 14. Setting of data collection | Where was the data collected? e.g. home, clinic, workplace  **Response:** Study setting has been described | Page 4 |
| 15. Presence of non-participants | Was anyone else present besides the participants and researchers?  **Response:** Non-study participants were not present*.* | page 4-5 |
| 16. Description of sample | What are the important characteristics of the sample? e.g. demographic data, date  Response: In the section of results, important characteristics of participants have been described including their gender, duration of hospital stay and the baby’s condition | Page 6 |
| *Data collection* |  |  |
| 17. Interview guide | Were questions, prompts, guides provided by the authors? Was it pilot tested?  **Response:** The interview guide had questions and probes. The interview guide was not piloted | page 5 |
| 18. Repeat interviews | Were repeat inter views carried out? If yes, how many?  **Response**: we did not have repeat interviews | Not applicable |
| 19. Audio/visual recording | Did the research use audio or visual recording to collect the data?  **Response:** The interview was audio recorded. | Page 5 |
| 20. Field notes | Were ﬁeld notes made during and/or after the inter view or focus group?  **Response:** We did not have field notes | Not applicable |
| 21. Duration | What was the duration of the inter views or focus group?  **Response:** Interviews lasted 18-37minutes. | Page 5 |
| 22. Data saturation | Was data saturation discussed?  **Response:** We used information power which was described in the section of sample size | Page 5 |
| 23. Transcripts returned | Were transcripts returned to participants for comment and/or correction?  **Response:** This was not done | Not applicable |
| **Domain 3: analysis and ﬁndings** |  |  |
| *Data analysis* |  |  |
| 24. Number of data coders | How many data coders coded the data?  **Response:** Two people were involved in coding | Page 5 |
| 25. Description of the coding tree | Did authors provide a description of the coding tree?  **Response:** We have provided table 1 to illustrate the coding tree | Page 5 and 7 |
| 26. Derivation of themes | Were themes identiﬁed in advance or derived from the data?  **Response**: The themes were derived inductively | Page 5 |
| 27. Software | What software, if applicable, was used to manage the data?  **Response:** Atlast ti software was used | Page 5 |
| 28. Participant checking | Did participants provide feedback on the ﬁndings?  **Response:** Participants did not provide feedback on the findings | Not applicable |
| *Reporting* |  |  |
| 29. Quotations presented | Were participant quotations presented to illustrate the themes/ﬁndings? Was each quotation identiﬁed? e.g. participant number  **Response:** Participants quotes were used to illustrate the findings. Each quote was identified using participant number | Page 6 to 17 |
| 30. Data and ﬁndings consistent | Was there consistency between the data presented and the ﬁndings?  **Response:** consistency was maintained between the data and the findings | Page 6 to 17 |
| 31. Clarity of major themes | Were major themes clearly presented in the ﬁndings?  **Response:** Major themes were clearly presented in the findings. | page 6 to 17 |
| 32. Clarity of minor themes | Is there a description of diverse cases or discussion of minor themes?  **Response:** Negative or diverse cases have been contrasted and discussed in the respective themes and subthemes | page 6 to 17 |
